# Boron Stress Activates the General Amino Acid Control Mechanism and Inhibits Protein Synthesis

**DOI:** 10.1371/journal.pone.0027772

**Published:** 2011-11-17

**Authors:** Irem Uluisik, Alaattin Kaya, Dmitri E. Fomenko, Huseyin C. Karakaya, Bradley A. Carlson, Vadim N. Gladyshev, Ahmet Koc

**Affiliations:** 1 Izmir Institute of Technology, Department of Molecular Biology and Genetics, Izmir, Turkey; 2 Division of Genetics, Department of Medicine, Brigham & Women's Hospital and Harvard Medical School, Boston, Massachusetts, United States of America; 3 Department of Biochemistry, University of Nebraska, Lincoln, Nebraska, United States of America; 4 Molecular Biology of Selenium Section, Laboratory of Cancer Prevention, Center for Cancer Research, National Cancer Institute, National Institutes of Health, Bethesda, Maryland, United States of America; Tulane University Health Sciences Center, United States of America

## Abstract

Boron is an essential micronutrient for plants, and it is beneficial for animals. However, at high concentrations boron is toxic to cells although the mechanism of this toxicity is not known. Atr1 has recently been identified as a boron efflux pump whose expression is upregulated in response to boron treatment. Here, we found that the expression of *ATR1* is associated with expression of genes involved in amino acid biosynthesis. These mechanisms are strictly controlled by the transcription factor Gcn4 in response to boron treatment. Further analyses have shown that boron impaired protein synthesis by promoting phosphorylation of eIF2α in a Gcn2 kinase dependent manner. The uncharged tRNA binding domain (HisRS) of Gcn2 is necessary for the phosphorylation of eIF2α in the presence of boron. We postulate that boron exerts its toxic effect through activation of the general amino acid control system and inhibition of protein synthesis. Since the general amino acid control pathway is conserved among eukaryotes, this mechanism of boron toxicity may be of general importance.

## Introduction

Boron is required for plant growth and development, and adequate levels of boron are crucial for high crop yield [1], [2], [3]. It has been established that the primary role of boron is the cross linking of cell wall components, rhamnogalacturonan-II and pectin, in plants [4]. Boron also forms complexes with glycoproteins in membrane structures [5] and it has been hypothesized that it is required for stabilization of molecules with *cis*-diol groups [6]. Apart from these structural functions, boron can interact with transcription factors and plays roles in cell signaling [7] and quorum sensing [8], [9]. This element also has beneficial effects for animal growth and development. It stimulates embryogenesis in zebrafish and trout [10], [11] and reduces the risk for prostate and lung cancers in humans [12], [13].

*Saccharomyces cerevisiae* emerged as a useful model to identify and characterize boron tolerance genes [14], [15], [16], [17], [18], [19]. *S. cerevisiae* is boron tolerant and can tolerate boric acid levels of up to 80 mM [14], [15]. Recently, the *ATR1* gene has been found to be a major boron tolerance gene [14]. Overexpression of *ATR1* provides high resistance to boron and reduces the intracellular boron levels. Mutants lacking the *ATR1* gene have been found to be sensitive to boron and have higher intracellular boron levels. Boron treatment has been shown to induce the *ATR1* gene and many amino acid biosynthesis genes that are normally regulated by the Gcn4 transcription factor [14].

In the case of amino acid starvation, yeast induces amino acid biosynthesis genes which are known as general amino acid control (GAAC) [20], [21], [22]. The Gcn4 transcription factor is the master regulator of GAAC that governs the concerted induction of a large group of biosynthesis genes [20], [22]. Interestingly, the expression of *GCN4* itself is regulated primarily at the translational level by four short open reading frames in its 5′-leader region [22], [23]. Activation of GAAC also triggers phosphorylation of the eukaryotic initiation factor–2 alpha (eIF2α) through the Gcn2 kinase and inhibits general translation [21], [24]. Activation of the Gcn2 kinase involves direct binding of uncharged tRNAs to the histidyl-tRNA synthetase domain of Gcn2 [21], [24], [25]. In yeast, eIF2α phosphorylation also leads to preferential translation of *GCN4* mRNA, while inhibiting general translation [21], [24].

In this study, we demonstrate that boron is sensed via its disruption of amino acid metabolism and translation. We postulate that induction of GAAC in response to boron stress is toxic because of its inhibitory effects on translation. However, GAAC-dependent *ATR1* activation is required for resistance to boron stress.

## Results

### Transcriptional Regulation of *ATR1*: Roles of the Gcn4 Transcription Factor in Boron Stress Response

**Figure 1:**
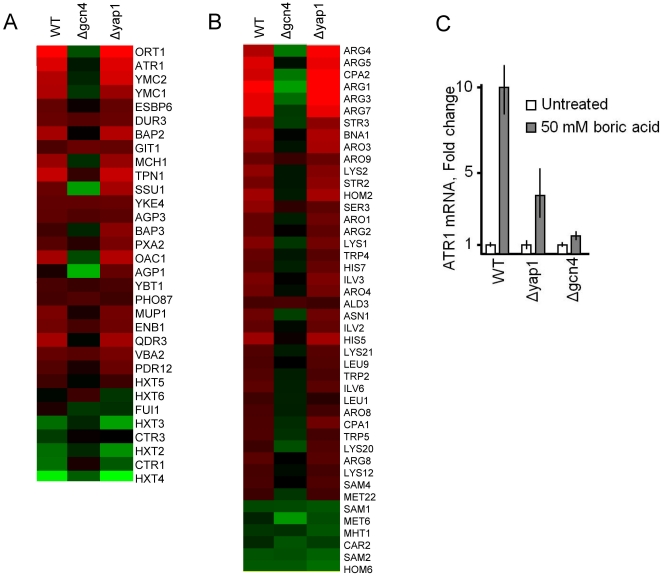
Gene expression analyses. To carry out microarray analyses, logarithmically growing wild type, *Δgcn4* and *Δyap1* mutant cells were split into two samples; one was treated with 20 mM boric acid for one hour while the other remained untreated. (A) Transporter genes that were upregulated or downregulated in response to boric acid treatment. (B) Amino acid biosynthesis genes that were upregulated and downregulated in response to boric acid treatment. Yeast *Saccharomyces cerevisiae* proteins annotation information from the yeast genome database (http://www.yeastgenome.org/ ) was used to identify transporters and other proteins. (C) Real time PCR analyses of *ATR1* in wild type, *Δgcn4* and *Δyap1* cells in response to boron. Cells were treated or not with 50 mM boric acid for one hour and *ATR1* expression was analyzed. Actin was used as the internal control. Bars represent the mean and SEM of triplicate assays.

Recently, Atr1 has been identified as a membrane transporter with boron efflux function. Treating cells with boric acid causes a four-fold increase in *ATR1* mRNA levels [14]. However, the factors that regulate *ATR1* in response to boron stress are not known. Previously, transcription factors Yap1 and Gcn4 were suggested to play a role in the regulation of *ATR1* [26]. Yap1 is a well known transcription factor that controls oxidative stress response genes and is likely to play a role in the activation of *ATR1* [27]. Gcn4 is the major regulator of gene expression during amino acid starvation and also under conditions of nutrient limitation in yeast [21]. Knowing that boron stress also induces amino acid biosynthetic genes [14], we reasoned that the Gcn4 transcription factor should regulate *ATR1* in response to boron treatment. To determine whether Yap1 or Gcn4 is required for *ATR1* induction by boron, global gene expression patterns of Δ*yap1* and Δ*gcn4* mutants were analyzed after boron treatment using DNA microarrays. The absence of *GCN4* prevented the activation of many transporters including *ATR1* **(** Fig. 1A **)** and amino acid biosynthesis genes (Fig. 1B), whereas the absence of *YAP1* had no effect on the expression of these genes. Thus, *ATR1* and the genes under the control of GAAC, such as amino acid biosynthesis genes and transporters, appear to be regulated by the transcription factor Gcn4 during boron stress. Gcn4 requirement for *ATR1* expression was also confirmed by a real time-PCR approach, in which *ATR1* mRNA levels were almost similar in control and mutant untreated cells, but upon boron treatment Δ*gcn4* mutants failed to induce *ATR1* transcription. However, deletion of *YAP1* partially prevented *ATR1* upregulation in response to boron (Fig. 1C).

Further processing of the data obtained by microarray analyses showed that boron treatment caused upregulation (≥2-fold) of 280 genes, and down regulation of 118 genes in wild type cells (Table S1, Table S2). However, many of the boron induced genes in wild type cells were not activated in *Δgcn4* mutants (Table S3, Figure 1A and 1B). Thus Gcn4 transcription factor has a central role in mediating boron stress response in yeast cells.

**Figure 2:**
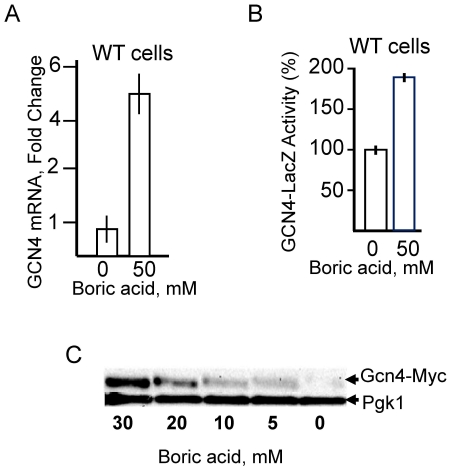
Induction of *GCN4* mRNA and protein expression by boric acid treatment. (A) Real time PCR analysis of *GCN4* mRNA in wild type cells treated with 50 mM boric acid for one hour. Actin was used as the internal control. (B) Wild type cells were transformed with the p180 plasmid which had the GCN4-lacZ reporter gene with all four uORFs of *GCN4*. Transformants were grown to logarithmic phase in YNB-URA medium. The cultures were split into two; one half was treated with 50 mM boric acid for one hour, and the other half remained untreated. β-galactosidase activities were measured as explained in Materials and Methods. (C) Effect of boric acid treatment on Gcn4 protein levels determined by western blotting. Chromosomally tagged GCN4-MYC cells were grown to logarithmic phase, treated with indicated amounts of boric acid and harvested (900 µl, 0.5 OD_600_). Primary (anti-c-myc) and secondary antibodies (anti-rabbit IgG) were used in a dilution of 1:3000 and 1:10000 respectively. The blot was stripped and reprobed yeast phosphoglycerate kinase monoclonal antibody as an internal control (1:1500 dilutions) and its 1:10000 diluted secondary antibody was goat anti mouse IgG conjugated with horseradish peroxidase.

Additionally, the microarray results showed that transcription of *GCN4* was 1.5-fold increased (p-value 2.8xE-3) in wild type cells treated with 20 mM boric acid for one hour, but none of the activators (*GCN1, GCN2, GCN3, GCN20*) of *GCN4* was upregulated (Table S1). The induction of internal *GCN4* expression in response to boric acid treatment was further analyzed by real time PCR (Fig. 2A). This assay confirmed that *GCN4* transcription is upregulated by boron and revealed that higher concentration of boron treatment (50 mM) causes a higher level of induction (5-fold). Since *GCN4* is mainly controlled at the translational level [21], we also tested whether boron stress increases the amount of Gcn4 protein in cells. As seen in Fig. 2B **,** boron treatment leads to almost two-fold induction of a chromosomal GCN4-dependent LacZ reporter gene activity in wild type cells. Increased protein level of *GCN4* was also shown by a western blotting approach using native *GCN4* gene tagged by MYC epitope (Fig. 2C).

**Figure 3:**
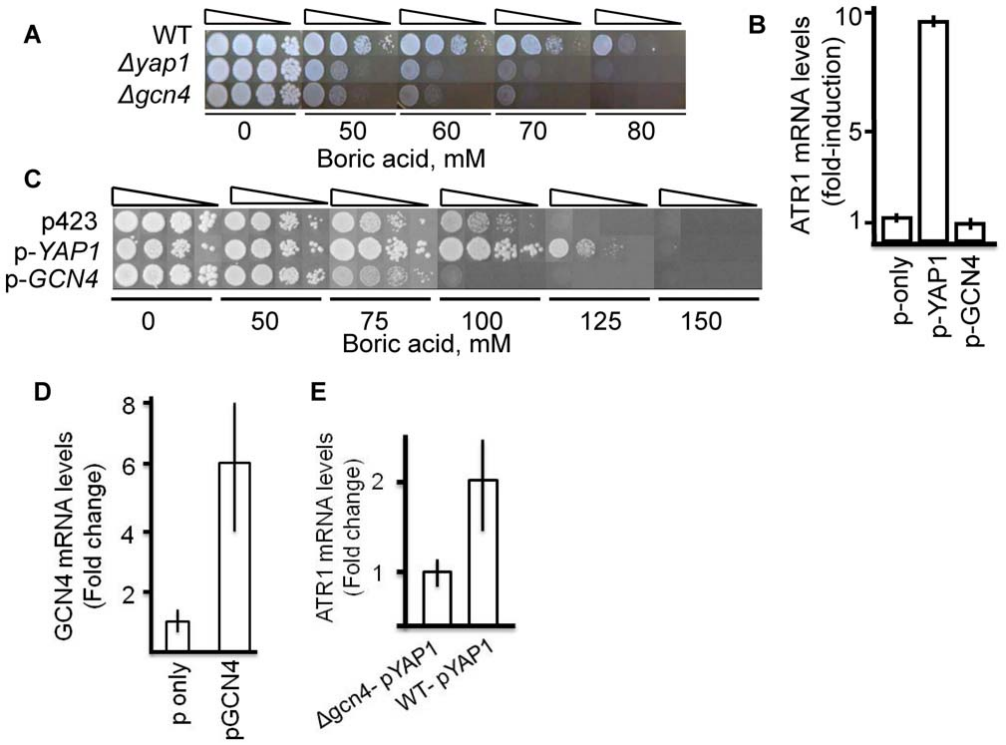
Effects of *GCN4* and *YAP1* overexpression on *ATR1* mRNA levels and boron tolerance. (A) Overnight cultures of wild type and isogenic *Δyap1*, *Δgcn4* yeast cells were first diluted to an OD_600_ of 0.2, 0.02, 0.002 and 0.0002, then 5 µl of each cell suspension were spotted onto YPD plates which contained different amounts of boric acid, as indicated. The cells were grown for 3 days at 30°C and photographed. (B) *ATR1* mRNA expression levels of wild type cells containing empty pAG423GPD vector, pAG423GPD-YAP1 and pAG423GPD-GCN4 vectors, respectively. Actin was used as an internal control. These experiments were performed three times. (C) Boric acid tolerance level of the same cells. The transformants were grown to logarithmic phase in YNB-HIS media, serially diluted to OD_600_ of 0.2, 0.02, 0.002, and 0.0002 and 5 µl of cell suspension were spotted onto plates containing the indicated amounts of boric acid. The plates were incubated at 30°C for 5 days and photographed. (D) *GCN4* mRNA expression levels in wild type cells overexpressing pAG423GPD and pAG423GPD-GCN4 vectors. (E) *ATR1* mRNA levels in wild type and *Δgcn4* mutant cells containing pAG423-YAP1 vector. Actin was used as an internal control.

To determine whether deletion of *GCN4* or *YAP1* renders cells boron sensitive, both mutants were inoculated to boron containing media and cell growth was followed. As seen in Fig. 3A, deletion of both of these genes sensitized cells to boron, suggesting that both Yap1 and Gcn4 might play roles in boron stress response.

It was previously shown that Gcn4 overexpression has toxic effects on yeast cell growth [28], [29]. We next overexpressed *GCN4* in wild type cells from a high copy plasmid, to test whether or not its expression activates transcription of *ATR1,* and in this manner provides boron resistance or not. As seen in Fig. 3B, *GCN4* overexpression in wild type cells neither increased the transcript level of *ATR1* nor led to boron resistance (Fig. 3C), although the overall *GCN4* level was notably elevated in wild type cells overexpressing *GCN4* (Fig. 3D).

Even though deletion of *YAP1* did not inhibit *ATR1* expression in response to boron stress, overexpression of *YAP1* from a plasmid stimulated the expression of *ATR1* and provided increased boron resistance (Fig. 3B and 3C). Overexpression of *GCN4* in Δ*gcn4* mutants showed effects similar to those in wild type cells (Fig. S1).

It was previously shown that Yap1 and Gcn4 bind to the same region of the *ATR1* promoter and regulate transcription in response to DNA-damaging agent 4-NQO [26]. To test whether Gcn4 is required for Yap1-dependent expression of *ATR1,* we overexpressed *YAP1* in Δ*gcn4* cells and observed a reduction in the level of *ATR1* transcript compared to that in wild type cells, suggesting that Yap1 requires Gcn4 to induce *ATR1* (Fig. 3E).

Overall, our gene expression analyses suggested that Gcn4 is required for *ATR1* induction and that it serves as a physiologically relevant transcription factor in the response to boron treatment. However, Yap1 could also induce *ATR1* and offer boron resistance when expressed ectopically.

### Boron Inhibits Protein Synthesis

**Figure 4:**
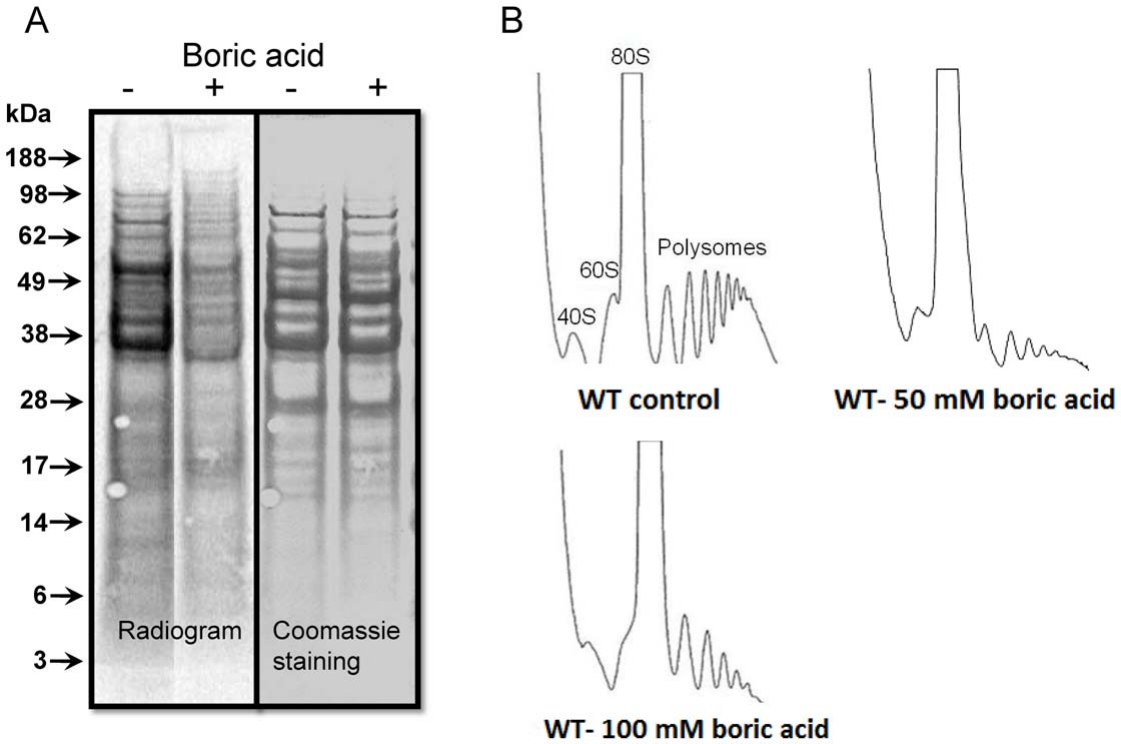
Protein synthesis is inhibited by boron. (A) Radiogram (left panel) showing incorporation of radioactive methionine into newly synthesized proteins. Coomassie staining of the same gel is shown in the right panel as a loading control. (B) Boron treatment decreases the abundance of polysomes. Wild type cells were grown to logarithmic phase and then treated with 50 and 100 mM of boric acid or left untreated. The cell lysates were analyzed by sucrose density gradient centrifugation. The absorbance profiles at 254 nm show 40S, 60S, 80S and polysome fractions, as indicated.

The activation of GAAC in response to boron stress prompted us to investigate the status of translation because Gcn2 kinase has a role in inhibiting protein synthesis upon activation [21]. Wild type cells were treated with 20 mM boric acid and labeled with radioactively labeled methionine for one hour. As shown in Fig. 4, boron-treated cells contained approximately 30% less ^35^S-labeled proteins than untreated cells (Fig. 4A, left panel) suggesting that boron inhibits protein synthesis. Translational status of boron-treated cells was further analyzed by the distribution of polysomes. Polysomes are clusters of ribosomes bound to an mRNA molecule that actively translates the message. They can be fractionated and quantified using sucrose density gradients. Cells that are grown in the absence of boron stress exhibit normal polysome profiles including peaks of 40S and 60S ribosomal subunits, 80S monosomes, and polysomes. After treatment with boric acid for one hour, we observed a dramatic shift from polysomal to monosomal states, suggesting that translation initiation collapses in the presence of boron (Fig. 4B). Inhibition of translation in response to certain stress conditions has been reported previously. For example, a recent study has shown that drugs generating oxidative stress as well as heavy metals stall initiation of translation in yeast [30].

### Boron Stress Induces Gcn2-dependent Phosphorylation of eIF2α

**Figure 5:**
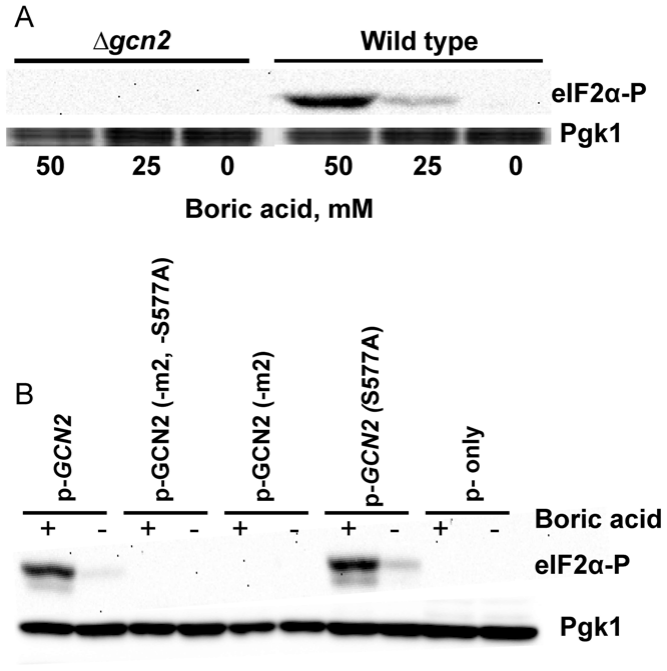
eIF2α is phosphorylated by boron treatment. (A) Wild type and *Δgcn2* cells were grown to logarithmic phase in YPD medium; the cultures were split and treated with 25 mM or 50 mM boric acid for one hour or left untreated. The levels of eIF2α phosphorylation at Ser51 were measured by western blot analysis using an antibody against phospho Ser51 on eIF2α. The membrane was stripped and reprobed with antibodies against yeast phosphoglycerate kinase (Pgk) antibody as an internal control. (B) Effect of boric acid on eIF2α phosphorylation depends on uncharged tRNA binding to an intact HisRS-like domain in Gcn2. The *Δ*gcn2 transformants carrying plasmids for an empty vector (p-only), GCN2 with S577A substitution (p-GCN2-S577A), GCN2 without HisRS domain (p-GCN2-m2), GCN2 without HisRS domain and with S577A substitution (p-GCN2-S577A-m2), and intact GCN2 (p-GCN2) were grown to logarithmic phase in YNB-URA medium and split in half; one of which was treated with 50 mM boric acid for one hour while the other was left untreated. Western blot analyses were performed with cell extracts using an antibody against eIF2α phosphorylated on Ser51. The membrane was stripped and reprobed with antibodies against yeast phosphoglycerate kinase (Pgk) as an internal control.

The protein kinase Gcn2 can be activated by a variety of conditions and it is the only known kinase in yeast that can phosphorylate eIF2α, which is responsible for carrying methionyl initiator tRNA (Met-tRNAi^Met^) to the 40S ribosomal subunit during the initiation stage of translation. Phosphorylation of eIF2α at serine 51 prevents GTP exchange by eIF2B and precludes the formation of a ternary complex for translation initiation, thus blocking protein synthesis. To determine whether the inhibition of protein synthesis in response to boron stress is dependent on the phosphorylation of eIF2α by Gcn2 kinase, wild type and Δ*gcn2* cells were treated with boron and the samples were subjected to western blotting analyses using an antibody which specifically recognizes the phosphorylated form of Ser51 in eIF2α. As seen in Fig. 5A, eIF2α is phosphorylated in wild type cells upon boron treatment, but not in Δ*gcn2* mutants. Thus, boron leads to phosphorylation of eIF2α in a Gcn2-dependent manner.

Apart from its kinase domain, Gcn2 contains a region similar to histidyl-tRNA synthetase (HisRS) which regulates Gcn2 kinase activity by monitoring uncharged tRNA levels in cells [25], [31]. Gcn2 activity can also be regulated by dephosphorylation of Ser577 [21]. To investigate whether Gcn2 activation is the result of uncharged tRNA binding or dephosphorylation of Ser577 in response to boron stress, we transformed Δ*gcn2* mutants with plasmids containing intact and mutated forms of the *GCN2* gene (*GCN2* without m2 domain [32], *GCN2* with S577A substitution [33], or a combination of both). The ability of transformants to phosphorylate eIF2α was investigated in boron-treated cells. As seen in Fig. 5B, eIF2α phoshorylation was unaffected by S577A substitution. However, mutation in the HisRS domain of Gcn2 (GCN2-m2), which abolishes uncharged tRNA binding to Gcn2, prevents eIF2α phosphorylation. Thus, the HisRS-like region is responsible for induction of Gcn2 kinase activity and this finding confirms that boron induces the uncharged tRNA signaling pathway.

### Boron inhibits aminoacylation of tRNAs

To determine whether boron inhibits the aminoacylation of tRNAs, we aminoacylated total calf liver tRNA with either ^3^H-serine or ^3^H-phenylalanine in the presence of various concentrations of neutralized boric acid. We found that boric acid inhibits both tRNA^Ser^ and tRNA^Phe^ formation if there is more than 50 mM boric acid in the reaction (Fig. S2). Thus, boron interferes with aminoacylation steps of the tRNAs and causes uncharged tRNA stress.

## Discussion

The essentiality of boron for plant growth is predominantly due to its presence in cell walls as a structural component of the rhamnogalacturonan II complex [4]. There has been no convincing evidence that boron toxicity is exerted through disruption of any process for which boron is required, notably via its role in cell walls. We recently suggested a potential link between translational control and boron toxicity in yeast based on the observation that mutants which show boron resistance were all lacking genes that play a role in uridine base modifications at the wobble position of tRNAs during translation [19]. Here, we found that the transcriptional activation of *ATR1* is mediated by the transcription factor Gcn4 in response to boron treatment. Further analyses have shown that boron activates the Gcn2 kinase, and leads to phosphorylation of eIF2α and inhibition of protein synthesis. The uncharged tRNA binding site (HisRS domain) of the Gcn2 kinase is required for Gcn2 to phosphorylate eIF2α. The requirement for HisRS-like domain (uncharged tRNA binding region) to phosphorylate eIF2α suggests that the Gcn2 kinase activity is stimulated by uncharged tRNAs. Eventhough, boron impairs the acylation of tRNAs, the mechanism by which boric acid interferes with tRNA acylation should be further investigated. Boric acid is a relatively unreactive compound, however, it can form ester bonds with several metabolites with multiple hydroxyl groups in the cis-configuration [6], [34]. Among such metabolites, one of the most important moieties is the ribose sugar found in ribonucleotides. It is likely that boric acid can bind to RNA species and interfere with the acylation reaction of tRNAs. In opposition to this, a recent study has shown that oxidative stress leads to degradation of tRNAs, mediated by Rny1 nuclease and induction of the Gcn2 kinase [35], [36]. We argue against this possibility since yeast cells lacking the *RNY1* gene still show a wild type level of eIF2α phosphorylation in response to boron treatment (data not shown).

It is also unclear how boron induces the transcription of Gcn4. Many stress and signaling pathways converge on the Gcn4 transcription factor and there are more than a dozen transcription factor binding sites in the *GCN4* promoter region [37]. Thus, further studies are required to identify upstream players in the boron stress response pathway.

Our results suggest a role for GAAC in boron stress response. It is known that several other stress conditions also activate GAAC. Among these stressors, transcriptional response to hydrogen peroxide [30], 3-AT and rapamycin [38] have also been analyzed in Δ*gcn4* cells and their datasets are publicly available. In order to understand whether the cellular response to boron stress is mediated via a unique or a common mechanism, we compared our microarray data to those of hydrogen peroxide, 3-AT and rapamycin treated cells. We also compared expression profiles of Δ*gcn4* mutants exposed to boron and other stress conditions to determine how Gcn4-dependent boron regulon differs from other regulons. We observed that the transcriptional response of wild type cells to boron and other stress conditions is quite different (Fig. S3). The major gene clusters, up- or down-regulated in response to boron treatment, did not show similarity to the patterns observed for 3-AT, rapamycin or hydrogen peroxide treatments. However, it is clear that the expression profiles of 3-AT and rapamycin treated cells share some common patterns. The expression profiles of Δ*gcn4* mutants exposed to boron and other stressors are also different (Fig. S3). This data clearly shows that the Gcn4-dependent boron regulon is different from those of other stress conditions and that boron stress has specific effects on the transcriptome.

Recently, it was shown that amino acid starvation in response to 3-AT treatment leads to repression of genes encoding ribosomal proteins via the transcription factor Gcn4 [39]. Our results showed that boron treatment does not change the expression pattern of most of the ribosomal protein genes, and that Gcn4 has no roles in regulating either *RPL* or *RPS* genes in response to boron stress (Table S1).

Overall, our results suggest that the general amino acid control pathway contributes to boron toxicity and transcriptional regulation of *ATR1* in response to boron stress. In this respect, we propose a model, wherein Gcn2 is activated by boron in an uncharged tRNA binding domain-dependent manner, activates Gcn4 and inhibits protein synthesis. Upon activation, Gcn4 induces the transcription of its boron specific target genes, *ATR1*, and many genes under the control of GAAC. Since the Gcn4-specific boron regulon shows differences with the activation of Gcn4 by other stress conditions, we speculate that there are other transcription factors that are involved in boron stress specific gene induction.

## Materials and Methods

### Yeast Growth, Media and Transformation

WT strain BY4741 (*MATa* his3 leu2 met15 ura3) and its isogenic deletion mutants were obtained from the yeast deletion library (Invitrogen). Cells were grown in either YEPD (2% glucose, 1% yeast extract, 2% peptone and 2% agar for solid media) or YNB (yeast nitrogen base) media supplemented with required amino acids and bases. Yeast transformations were performed using the LiAc method [40].

### DNA Microarray Analysis

BY4741 wild type, Δ*yap1*, Δ*gcn4* mutant cells were grown to an optical density of 0.2–0.3 at 600 nm in 200 ml YPD medium, with or without 20 mM boric acid treatment for 1 hour. Cells were then harvested by centrifugation. Total RNA was isolated using the Ambion RiboPure™-Yeast Kit. The Agilent Two-Color Low RNA Input Linear Amplification Kit was used to generate fluorescently labeled cRNA for two-color microarray hybridizations. Fluorescently labeled cRNA molecules were purified from the reaction mixture using the Qiagen RNeasy mini kit. The resulting cRNA samples (825 ng each) were mixed with Agilent Hi-RPM hybridization buffer. Microarray hybridizations were performed using Agilent SureHyb hybridization chambers. The hybridization chambers were loaded onto a rotisserie in an Agilent Hybridization oven and were incubated at 65°C for 17 hours with a rotational speed of 10 rpm. Following incubation, the microarray slides were washed for 1 min each in Gene Expression wash buffer 1 (6×SSPE, 0.005% N-lauroylsarcosine, at room temperature) and Gene Expression Wash Buffer 2 (0.06×SSPE, 0.005% N-lauroylsarcosine, at 31°C) for 1 min each. The microarray slides were briefly dipped in a solution of acetonitrile, dried and scanned in an Agilent Technologies G2505B microarray scanner at 5 µm resolution. Microarray data is MIAME compliant; the raw data has been deposited in the GEO database (Accession number GSE29887).

### Western Blot Analyses

Wild type, and the isogenic *Δgcn2* and *GCN4-MYC* strains were grown to mid-log phase in either YPD or selective YNB-Ura media, with or without boron treatment and equal numbers of cells were harvested by centrifugation for each strain. Cell extracts were resuspended in sample buffer (0.06 M Tris-HCl, pH 6.8, 10% (v/v) glycerol, 2% (w/v) SDS, 5% (v/v) 2-mercaptoethanol, 0.0025% (w/v) bromophenol blue) and heated at 95°C for 5 min. Samples were separated in 5–15% SDS-PAGE and electrotransferred to a PVDF (Sigma) membrane. Primary antibodies against phospho (Ser51) specific eIF2α were acquired from USBiological and anti-c-myc antibody used for detection of *GCN4-MYC* was from Sigma. The blots were stripped and reprobed with monoclonal antibodies specific for yeast phosphoglycerate kinase (Invitrogen) as an internal control. As secondary antibodies, anti-rabbit IgG conjugated to horseradish peroxidase (Sigma) or goat anti mouse IgG conjugated to horseradish peroxidase (Sigma) were used. The membranes were treated with enhanced chemiluminescence system (Bio-Rad) and visualized using the VersaDoc Imaging System (Bio-Rad).

### Metabolic Labeling

BY4741 yeast cells were grown in 200 ml of YNB medium to OD_600_ = 0.5, split to 100 ml aliquots, and one aliquot was treated with 20 mM of boric acid. After 1 hour of incubation, both aliquots were metabolically labeled with 0.5 mCi of EXPRE35S35S Protein Labeling Mix (Perkin-Elmer) for 30 min. Cells were harvested by centrifugation, washed three times with PBS buffer and disrupted with glass beads for 10 min in PBS buffer. The resulting cellular lysates were normalized according to protein concentration and 10 µg of each lysate was subjected to SDS-PAGE. Resolved proteins were transferred onto PVDF membrane. ^35^S-labeled proteins were visualized with a Storm PhosphorImager system (Molecular Dynamics). ImageQuant package was used to quantify the autoradiogram data.

### Polysome Profiling

Density gradient centrifugation system was used in order to separate total RNA based on the molecular weight. In brief, 5% and 70% sucrose solutions [RiboLock™ RNase inhibitor (Fermentas), 100 mM NaCl, 10 mM MgCl_2_, 20 mM HEPES] were prepared and thereafter sucrose gradients were prepared by using ISCO systems. BY4741 cells were grown to an optical density of 0.7–0.9 at 600 nm in 30 ml YPD medium. These cells were either exposed to 50 mM or 100 mM boric acid for 1 hour or not exposed. They were harvested by centrifugation, washed twice with distilled water, and frozen at −80°C. Then, yeast cells were homogenized in 5 ml lysis buffer [100 mM NaCl, 10 mM MgCl_2_, 20 mM HEPES, 40 u/µl RiboLock™ RNase inhibitor (Fermentas), 1% Triton-X 100, 1% sodium deoxycolate (NaDOC), 10 mg/ml cycloheximide (Sigma)] by vortexing with glass beads for 10 min. After ten minutes of incubation on ice, homogenates were centrifuged for 10 min at 12,000 g and 4°C. The supernatants were transferred to 5%–70% (w/v) sucrose gradients and sedimented for 2.5 h, at 27,000 rpm, 4°C, in an SW28 rotor using a Beckman Coulter Optima™ L-XP Ultracentrifuge System. Forty fractions were collected from the top of the gradient by using an ISCO Tris pump while monitoring absorbance at 254 nm with an ISCO UA-6 monitor. The RNP populations were grouped according to their sedimentation rates: mRNP, 40S, monosomes and polyribosome fractions.

### Boron Tolerance Assays

YPD (2% glucose, 2% peptone, 1% yeast extract and 2% agar) or YNB (2% glucose, 0.67% yeast nitrogen base without amino acids and 2% agar) agar plates with different concentrations of boric acid were prepared. Overnight cultures were diluted to an optical density of 0.2 at 600 nm and then serially diluted to 0.02, 0.002, and 0,002. A 5 µl aliquot of each dilution was spotted on plates and incubated for 3 days at 30°C.

### RNA Isolation and Real Time PCR Analyses

Total RNA was isolated from boron-treated and untreated samples using an RNA isolation kit (Invitrogen). Genomic DNA traces were removed by DNAse treatment (Fermentas). Complementary DNA (cDNA) from total mRNA was obtained using the First Strand cDNA Synthesis Kit (Fermentas). The cDNA was used as a template for amplifying a 186 bp fragment for the *ATR1* gene, a 155 bp fragment for the *GCN4* gene and a 103 bp fragment for the internal control *ACT1* gene. Triplicate real-time PCR analyses were performed with the IQ5 real-time PCR system (Bio-Rad) using Fermentas Maxima^TM^ SYBR Green qPCR Master Mix (2X). The primers used for the amplification of *ATR1* were ATR1F (5′-ACGCGTATAGCATAGCCGCTTTCA-3′) and ATR1B (5′-TGTAAGCCTGGTTCCAACCCGATA-3′); the primers used for the amplification of *GCN4* were GCN4F (5′-ATCCAATGGGTTTCTCACCA-3′) and GCN4B (5′- CGAAGGGGTATCCTGTTTGA-3′); and those used for the amplification of *ACT1* were ACT1F (5′-ACGTTCCAGCCTTCTACGTTTCCA-3′) and ACT1B (5′-ACGTGAGTAACACCATCACCGGAA-3′). The conditions of PCR amplification for *ATR1* were as follows: 40 cycles at 94°C for 30 s, 58°C for 30 s and 72 °C for 30 s, and for GCN4: 40 cycles at 94°C for 30 s, 56°C for 30 s, and 72°C for 30 s.

### Cloning of YAP1 and GCN4 Genes

Wild type alleles of *YAP1* and *GCN4* were amplified using High Fidelity Enzyme (Fermentas) from yeast genomic DNA using the following primers: YAP1F (5′GGGGACAAGTTTGTACAAAAAAGCAGGCTCTTAAACCATGAGTGTGTCT-3′) and YAP1B (5′GGGGACCACACTTTGTACAAGAAAGCTGGGTGTTCCCGCTTTAGTTCATAT-3′) for *YAP1*, and GCN4F (5′GGGGACAAGTTTGTACAAAAAAGCAGGCTAATGTCCGAATATCAGCCAA-3′) and GCN4B (5′GGGGACCACTTTGTACAAGAAAGCTGGGTATGAAATCAGCGTTCGCCAA-3′) for *GCN4*. The amplified fragments were first cloned into the pDONR vector using 2 µl BP Clonase^TM^ II enzyme (Invitrogen) with an overnight incubation at 25°C and then moved to LR clonase reaction. This reaction was performed with 2 µl of isolated entry clones, 1.5 µl pAG423GPD expression vector, 3 µl TE buffer and 2 µl LR Clonase^TM^ II enzyme mixture (Invitrogen). Isolated plasmids were verified by sequence analyses, and then yeast cells were transformed with the empty vector or with a gene expression construct, and selected for histidine prototrophy. Plasmids with Gcn2 (p4384, flag epitope-tagged form of *GCN2*; p4055, nonphosphorylatable S577-Alanine mutation; p4056, substitutions in the m2 motif of the HisRS-like domain; p4057, with substitutions both in the m2 motif and the S577A motif) were obtained from Alan Hinnebusch (NIH).

### β-galactosidase activity assays

Wild type cells were transformed with the p180 plasmid which carries the GCN4-lacZ gene [41]. Overnight cultures of wild type cells in 25 ml of YNB-URA were diluted to 0.1 at OD_600_. After 2 hours incubation, cultures were split into two flasks with or without 50 mM boric acid. After 1 hour growth in the presence of boron, 100 µl of cell solution, at an optical density between 0.2 and 0.6 at 600 nm, was taken and homogenized in 400 µl of Z-buffer [60 mM Na_2_HPO_4_, 40 mM NaH_2_PO_4_, 10 mM KCl, 1 mM MgSO_4_, 50 mM β-mercaptoethanol and 0.2% N-lauryl sarcosine]. After 30 min preincubation at 30°C, 150 µl of Z-buffer with 4 mg/ml, ONPG (o-nitrophenyl-β-D-galactopyranoside) (FlukaBioChemika) was added to the suspensions and the resulting solutions were incubated at 30°C for one hour. The reaction was stopped by adding 400 µl 1.5 M Na_2_CO_3_. After centrifugation for 1 min, supernatants were taken and their absorbance assayed at 420 nm [42]. The obtained absorbance values were normalized according to the OD values of cells.

### Aminoacylation of tRNAs in boric acid

Total calf liver tRNA (Sigma) was aminoacylated under limiting tRNA conditions with either ^3^H-serine (Moravek Biochemicals, specific activity 20 Ci/mmol) or ^3^H-phenylalanine (GE Healthcare, specific activity 123 Ci/mmol) and 19 unlabelled amino acids in the presence of rabbit reticulocyte synthetases as described [43] and various concentrations of boric acid (Sigma).

## Supporting Information

Figure S1 **Growth rates of cells transfromed with GCN4 or YAP1 plasmids.**
(TIFF)

Figure S2 **Inhibition of aminoacylation of tRNAs by boric acid.**
(TIF)

Figure S3 **Gcn4 regulates different sets of genes in response to various stress conditions.** Comparison of genomic expression profiles in response to boric acid, rapamycin, 3-AT and H2O2 in wild type and *gcn4Δ* cells using publicly available datasets [38].
(TIF)

Table S1 **Gene expression profiles of WT, gcn4- and yap1- cells in response to 20 mM boric (1 h treatment).**
(XLS)

Table S2 **Genes whose expression changed more than 2-fold in response to boron stress in wild type cells.**
(XLS)

Table S3 **Genes whose expression changed more than 2-fold in gcn4Δ cells in response to boron.**
(XLS)
