# Supplementary figures and images for: Boron Stress Activates the General Amino Acid Control Mechanism and Inhibits Protein Synthesis
Source: PLoS One. 2011 Nov 17;6(11):e27772. doi: 10.1371/journal.pone.0027772 (PMC3219688; doi:10.1371/journal.pone.0027772)

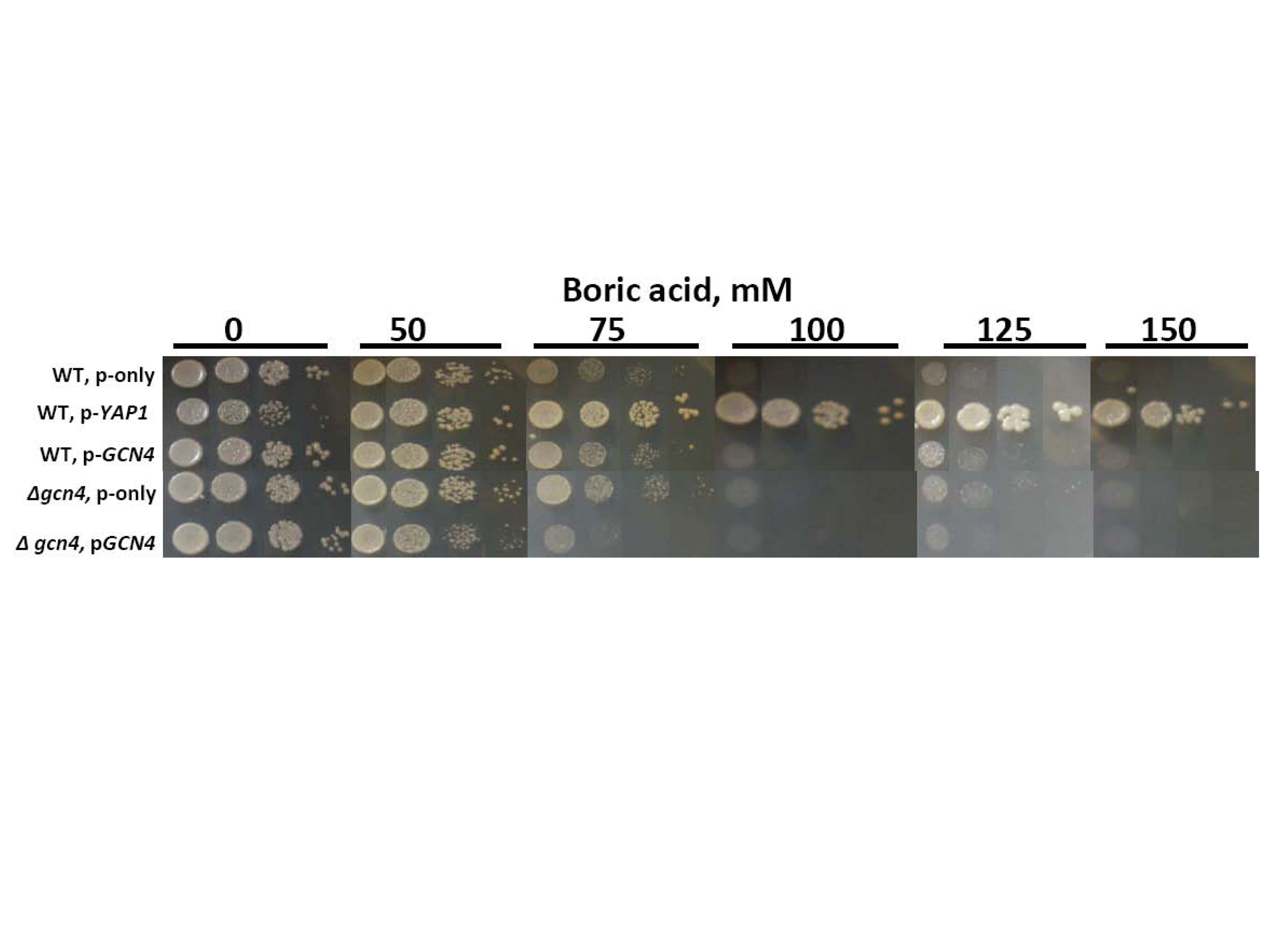

Supplement: Figure S1 — Growth rates of cells transfromed with GCN4 or YAP1 plasmids. (TIFF) [file pone.0027772.s001.tiff]

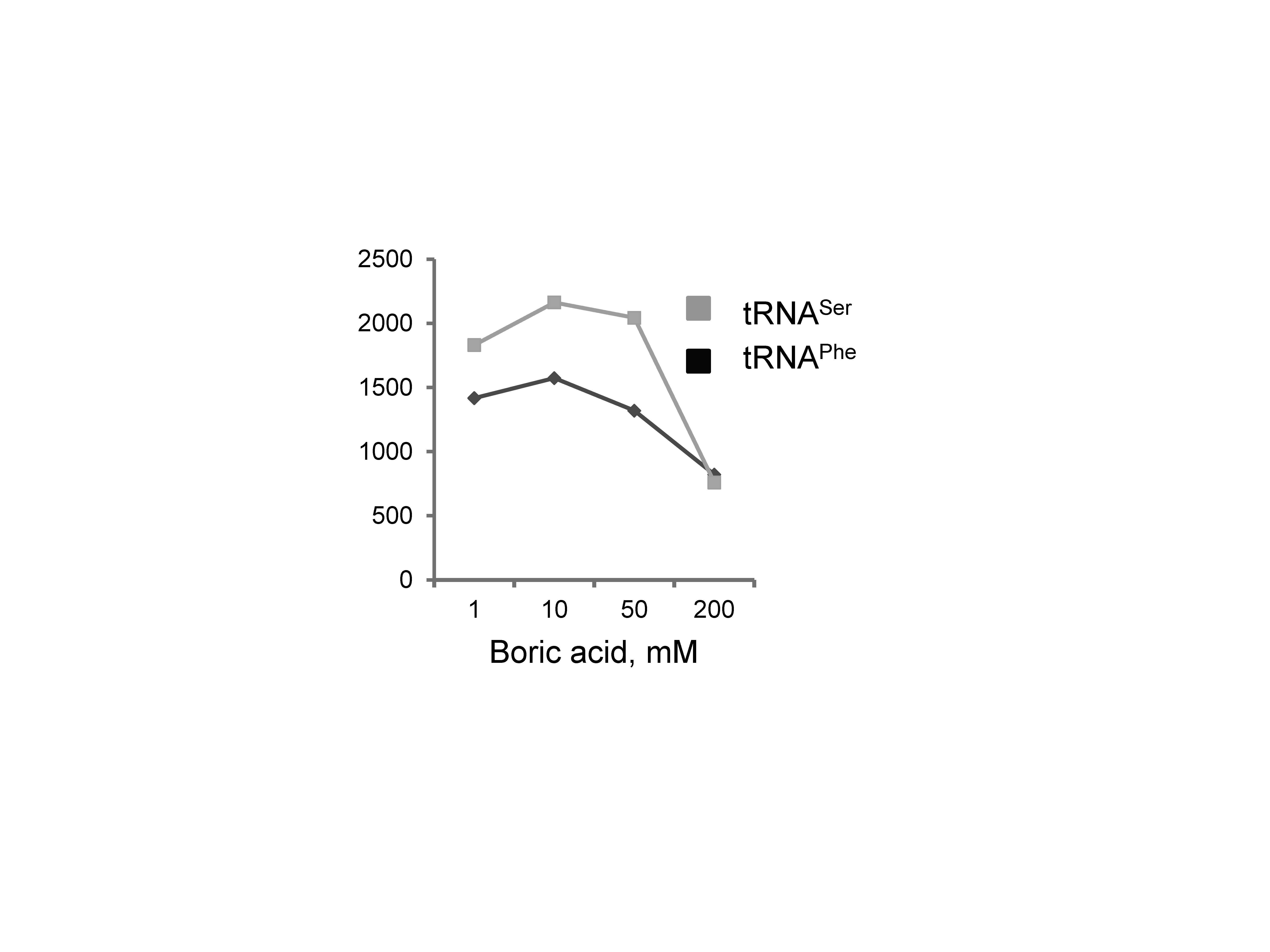

Supplement: Figure S2 — Inhibition of aminoacylation of tRNAs by boric acid. (TIF) [file pone.0027772.s002.tif]

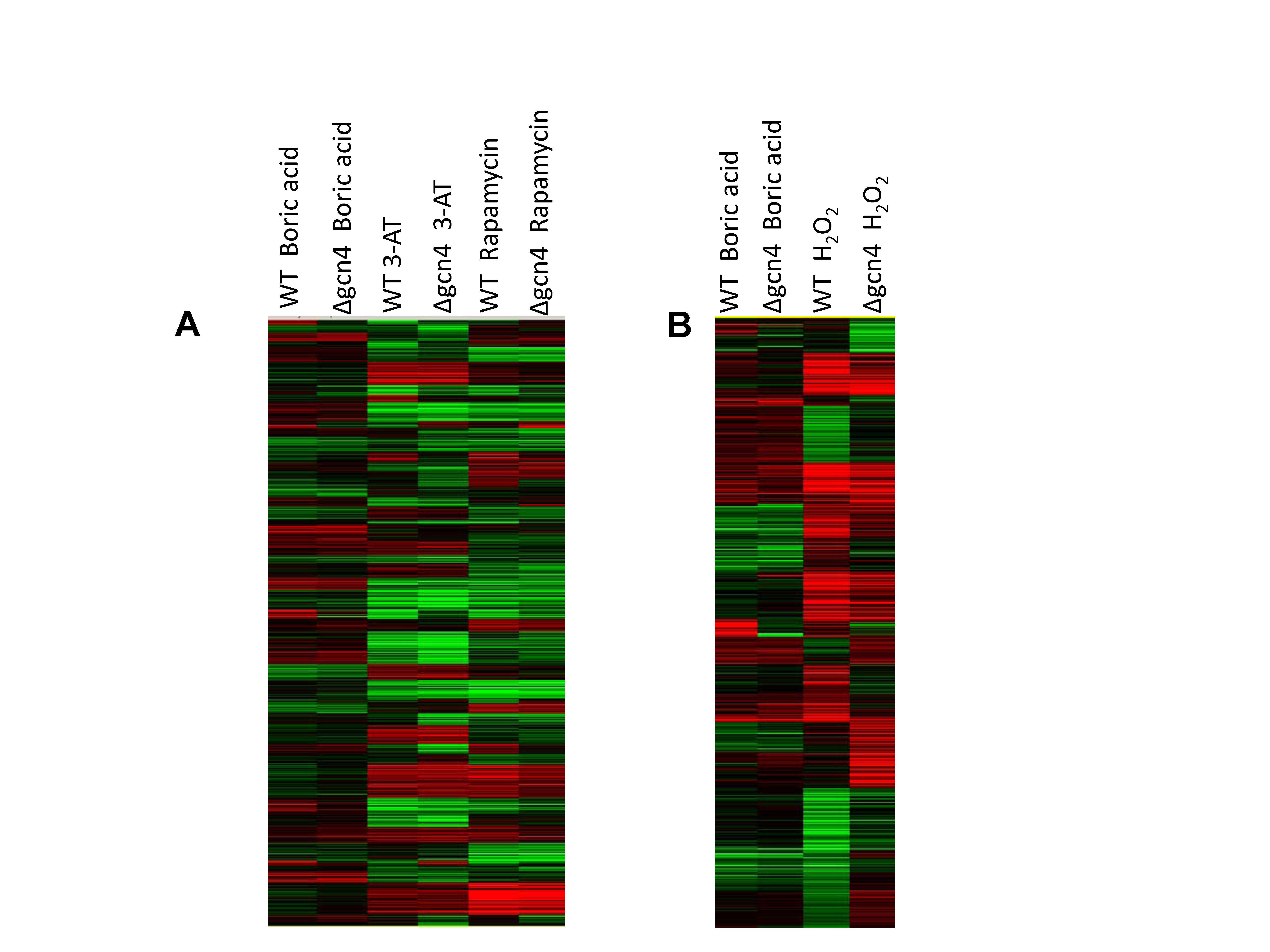

Supplement: Figure S3 — Gcn4 regulates different sets of genes in response to various stress conditions. Comparison of genomic expression profiles in response to boric acid, rapamycin, 3-AT and H2O2 in wild type and gcn4Δ cells using publicly available datasets [38]. (TIF) [file pone.0027772.s003.tif]
